# Trends in the Use of Common Words and Patient-Centric Language in the Titles of Medical Journals, 1976-2015

**DOI:** 10.1001/jamanetworkopen.2019.1083

**Published:** 2019-03-22

**Authors:** Gregory M. Chen, Sarshan R. Pather, Horace M. DeLisser

**Affiliations:** 1 Graduate Group in Genomics and Computational Biology, University of Pennsylvania, Philadelphia; 2 Medical Scientist Training Program, Perelman School of Medicine, University of Pennsylvania, Philadelphia; 3 Graduate Group in Cell and Molecular Biology, University of Pennsylvania, Philadelphia; 4 Division of Pulmonary, Allergy and Critical Care, Perelman School of Medicine, University of Pennsylvania, Philadelphia; 5 Academic Programs, Jordan Medical Education Center, Perelman School of Medicine, University of Pennsylvania, Philadelphia

## Abstract

**Importance:**

The language of medical research appears to be intrinsically tied to the culture of medical research and provides a unique window into broader trends in the culture of medicine.

**Objective:**

To analyze medical language from 5 premier medical journals and investigate broader changes in the culture of clinical investigation during the last 40 years.

**Design, Setting, and Participants:**

In this qualitative study using a data-driven analysis, 302 293 PubMed records were extracted from *JAMA*, *The Lancet*, *Annals of Internal Medicine*, the *BMJ*, and *New England Journal of Medicine* from January 1, 1976, through December 31, 2015, to identify key trends in medical language. A frequency analysis was applied across the 40-year time frame in *JAMA* to assess the major trends in all publication types. Patient-centered language was analyzed in clinical trials in the flanking time periods (1976-1980 and 2011-2015) across the 5 journals. Data were analyzed from November 16, 2016, through November 9, 2018.

**Main Outcomes and Measures:**

Increasing or decreasing frequency of words (monograms) and word pairs (bigrams) and the proportion of patient-centric words in journal article titles.

**Results:**

In *JAMA*, 50 277 articles of all publication types were included. In the frequency analysis, the most increased terms were reflective of the language of epidemiological research. The bigram analysis revealed a decline in causal language (−2.42/100 000 words to −2.03/100 000 words; false discovery rate [FDR], <0.01) and an increased description of patients in the plural form (6.92/100 000 words to 11.4/100 000 words; FDR, <0.01). A trend to separate patient from disease was observed; for example, there was a decrease in describing a patient as a *diabetic* (−2.21/100 000 words; FDR, <0.01) compared with *a patient with diabetes*. In the analysis of clinical trials in all 5 journals, 3125 titles were identified (range, 193-932 per journal). In 4 of the 5 journals, use of patient-centric keywords increased significantly (absolute increase, 18.9%-34.3%; *P* < .001 for 3 journals; *P* = .01 for 1 journal), with the *New England Journal of Medicine* as the exception. This finding reflects a change from shorter disease-centric titles to longer titles that describe patients with a disease.

**Conclusions and Relevance:**

Trends in medical language reflect the rise of evidence-based medicine, a shift in focus from individuals to populations, and a separation of patient and disease. Data-driven analysis of medical language provides a unique window into the changing landscape of medical culture.

## Key Points

**Question:**

Does the language of medicine in academic journals indicate whether the culture of clinical investigation has shifted toward patient centeredness?

**Findings:**

In this qualitative study of medical language of 302 293 articles from 5 premier medical journals, use in the last 40 years has changed to reflect a shift from individuals to populations, a separation of patient and disease, and an increase in patient-centric titles.

**Meaning:**

Whereas medical language previously emphasized treatments and disease processes, the trend during the last 40 years has been to separate patients from their disease and to emphasize the patient rather than characterize patients by their disease.

## Teaser

This qualitative study of medical research language assesses changes in 5 medical journals and broader changes in the culture of clinical investigation during the last 40 years.

## Introduction

The culture of clinical investigation has undergone dramatic changes in the past 40 years. The 1970s marked the high-profile termination of the Tuskegee Syphilis Study and subsequent drafting of the National Research Act and Belmont Report, which set new ethical and legal frameworks for human research.^1,2^ Central to these documents was an understanding of the dual view that physician-scientists must take to recognize patients as research participants and complex individuals. More recently, the rise of evidence-based medicine has encouraged higher standards of scientific rigor in clinical studies, favoring evidence from large randomized clinical trials over case reports and clinical experience. Evidence-based medicine has offered a cultural paradigm shift in not only the experimental design of medical research, but the central thought processes regarding patients, clinical decision making, and the origin of medical knowledge.^3,4^ Concurrent with these major movements, the physician-patient relationship has changed, emphasizing the importance of communication and favoring patient autonomy over paternalism.^5,6,7,8,9^

We sought to explore the changing culture of clinical investigative medicine through changes in the language of medicine. In using the term *culture*, we are referencing “an integrated pattern of learned beliefs and behaviors that can be shared among groups and include thoughts, styles of communicating, ways of interacting, views of roles and relationships, values, practices, and customs.”^10^^(p561)^ The relationship between medical culture and medical language is complex and bidirectional, with each influencing the other. We posit that the language of medicine in academic journals provides a unique window into broader changes in the culture of clinical investigation. We therefore sought to identify trends in word usage in major medical journals and assessed whether patient-centered language increased or decreased in reporting of clinical trials. Because clinical investigation has shifted from case reports to large trials, the potential exists for the language of clinical research to become distanced from patients and reflect a scientific enterprise in which patients fulfill the role of subjects in large multicenter experiments. In contrast, modern movements emphasizing patient autonomy and patient-centered care might have led to a broad cultural shift in which patients in clinical research are perceived as individuals in a patient-centered manner. To address these questions, we focused on the content of article titles, which reflect the key components that authors, reviewers, and editors perceive to be the most important elements of their communication. We applied a quantitative text-mining approach, extracting the titles of more than 300 000 articles in PubMed to identify key trends in the medical literature reflective of broader changes in medical culture.

## Methods

### Data Extraction

We extracted MEDLINE/PubMed records in XML format from all articles published in *JAMA*, *The Lancet*, *Annals of Internal Medicine*, the *BMJ*, and *New England Journal of Medicine* (*NEJM*) using National Center for Biotechnology Information Entrez Programming Utilities command-line tools.^11^ Approval of this study was waived by the institutional review board of the University of Pennsylvania because it did not meet the definition of human participant research. This study follows the Standards for Reporting Qualitative Research (SRQR) reporting guidelines.

### Identification of Increased and Decreased Words in *JAMA*

We focused initially on *JAMA* and parsed MEDLINE/PubMed XML elements to extract the title text, publication types, and date of publication for articles from January 1, 1976, through December 31, 2015. Title texts for all publication types were loaded as objects in R package tm,^12^ and we computed single-word (monogram) and word-pair (bigram) frequencies for each year in the range. Monograms and bigrams of very low frequency (<0.01%) within the 40-year range were excluded from the analyses. For the monogram analysis, stop words such as *the*, *to*, and *and* were removed. We identified significantly increased and decreased words and word pairs using a continuous linear model through the 40-year period. Visualization as a word cloud was performed using the R package wordcloud.^13^

### Assessment of Patient-Centric Nouns in Clinical Trial Reporting

Next, we sought to investigate the use of patient-centric nouns in clinical trials reported in *JAMA* in addition to the 4 other medical journals. For this analysis, articles were first included if they were indexed with a publication type of clinical trial of any phase, randomized clinical trial, pragmatic clinical trial, or controlled clinical trial. We then applied exclusion criteria if the article was indexed as any publication type in biography, case reports, classical article, clinical conference, comment, congresses, consensus development conference, duplicate publication, editorial, guideline, historical article, legal cases, letter, news, patient education, handout, portraits, retracted publication, or review. For the 5 years at the beginning and end of our study period (1976-1980 and 2011-2015), we compiled all articles of these publication types in *JAMA* and the 4 other journals. Subsequently, 2 independent raters (G.M.C. and S.R.P.), blinded to journal name and publication year, classified each title as patient centric or non–patient centric using a set of predefined criteria (eTable in the Supplement).

### Statistical Analysis

Data were analyzed from November 16, 2016, through November 9, 2018. For the word-frequency analysis in *JAMA* across all articles, we applied a linear model for each term, with monogram or bigram frequency as the dependent variable and the publication year as the independent variable. Significant changes in frequency were identified with the 2-tailed *t* test and Benjamini-Hochberg multiple testing correction with a false discovery rate (FDR) threshold of 0.01. For the analysis of patient-centered nouns in clinical trials, interrater agreement was assessed with the Cohen κ statistic, and 1 of us (H.M.D.) resolved any conflicts by casting the tie-breaking vote. We used a 2-sided 2-proportion *z* test to assess for differences in proportions between the early and late periods, and *P* < .05 was considered significant.

## Results

We extracted 302 293 articles of all publication types from all 5 journals in the complete 1976-2015 period, of which 50 277 were from *JAMA*. Applying our criteria for clinical trials in the ranges of 1976 to 1980 and 2011 to 2015, we included 3125 titles total, with 193 from *Annals of Internal Medicine*, 648 from *BMJ*, 476 from *JAMA*, 932 from *The Lancet*, and 876 from *NEJM*.

**Figure 1.**
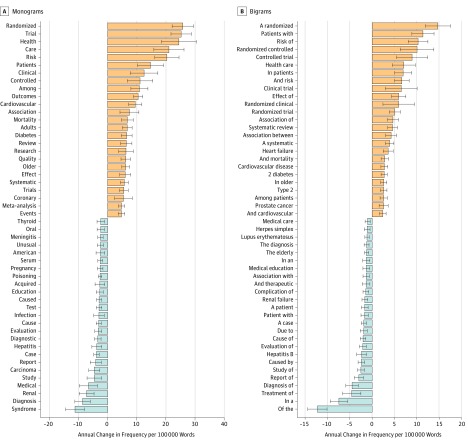
Bar Plots of Increased and Decreased Monograms and Bigrams in *JAMA* The top 20 increased and decreased monograms (single words) and bigrams (pairs of adjacent words) are given as mean (95% CI). For articles of all publication types published from 1976 through 2015, all monograms or bigrams were extracted, and a linear model was performed to estimate mean annual change in frequency during the 40-year period. Common words such as *the*, *to*, and *and* were removed from the monogram analysis, but kept in the bigram analysis owing to their contextual relevance.

**Figure 2.**
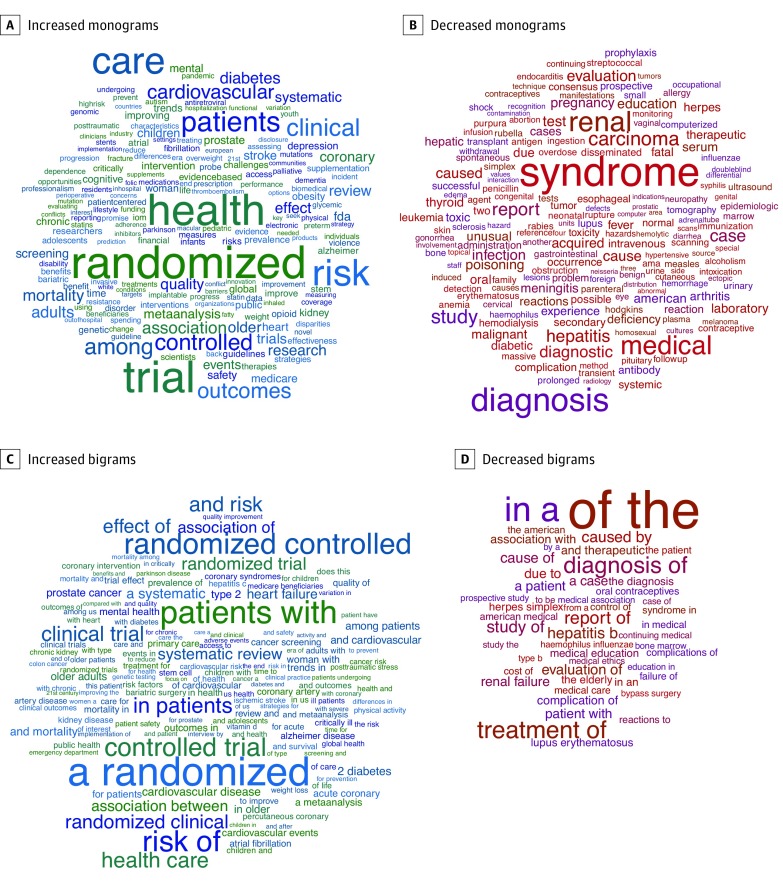
Word Cloud Representation of Significantly Increased or Decreased Monograms and Bigrams in *JAMA* During the 40-Year Period Monograms indicate single words; bigrams, pairs of adjacent words. Articles of all publication types published in *JAMA* from 1976 through 2015 were included. The size of each word or pair of words is proportionate to the magnitude of the absolute change in frequency during the course of the 40-year study period.

In *JAMA*, the most increased terms across our 40-year study period were reflective of the language of epidemiological research (Figure 1 and eFigure in the Supplement). Monograms (Figure 1A) such as *randomized*, *trial*, *outcomes*, and *risk* showed significant increases (mean annual frequency change per 100 000 words, 10.48 to 25.81; FDR < 0.01), consistent with a concurrent decrease in case reports and increase in clinical trial reports across this period. We also noted a shift toward referring to patients in the plural form: bigrams (Figure 1B) such as *a patient* (−1.66/100 000 words) and *patient with* (−1.68/100 000 words) decreased (FDR < 0.01) and *in patients* (6.92/100 000 words) and *patients with* (11.37/100 000 words) increased (FDR < 0.01). The use of *the elderly* decreased (−1.25/100 000 words; FDR < 0.01), whereas *older patients* (1.07/100 000 words) and *older adults* (2.38/100 000 words) increased (FDR < 0.01). We observed a general decline in the language of causality, with a decreased frequency of the bigrams *caused by* (−2.42/100 000 words) and *cause of* (−2.03/100 000 words) (FDR < 0.01) and a concurrent increase in *association between* (4.24/100 000 words) and *association of* (4.66/100 000 words) (FDR < 0.01). Although *diabetes* was among the top significantly increased monograms (6.57/100 000 words), the frequency of the word *diabetic* was significantly decreased in this time period (−2.21/100 000 words; FDR < 0.01). Word clouds were also produced for statistically significant increases or decreases in monograms or bigrams (Figure 2).

**Figure 3.**
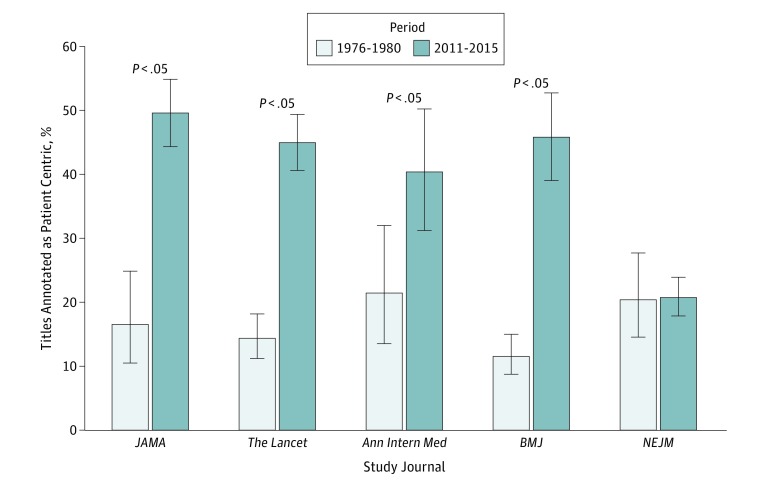
Proportions of Patient-Centric Titles in the 5 Study Journals A manual review was performed of titles of 3125 articles indexed as clinical trials and published from 1976 through 1980 and 2011 through 2015 in *JAMA*, *The Lancet*, *Annals of Internal Medicine* (*Ann Intern Med*), *BMJ*, and *New England Journal of Medicine* (*NEJM*). Error bars indicate 95% CIs. The eTable in the Supplement gives inclusion and exclusion criteria for defining the presence of a patient-centric noun.

Using predetermined criteria (eTable in the Supplement), we assessed whether a separation between patient and disease was also found in 4 other premier journals. Interrater agreement for article annotation was high (Cohen κ = 0.89). In 4 of 5 journals, we observed a significant increase in the use of patient-centric titles (absolute percentage increase: 18.9%-34.3%; *P* < .01 for *Annals of Internal Medicine* and *P* < .001 for others), with the exception of *NEJM* (Figure 3). In addition, the mean title length significantly increased for 4 of the 5 journals (mean increase in character count: 38.3-81.6; *P* < .001), except *NEJM*, in which mean title length decreased (mean decrease in character count, 20.6; *P* < .001). Together these data point to a change from shorter disease-centric titles to longer titles that emphasize patients with a disease.

## Discussion

Given the growth in annual publication of biomedical and clinical research articles, text-mining approaches provide a unique opportunity to investigate broader cultural trends in medicine and science. Similar approaches have been applied to uncover novel information hidden in complex high-volume data sets in the biomedical domain, ranging from systematic searches for biomarkers for drug discovery to the extraction of useful data to augment clinical decision making and diagnostic strategies for patient care.^14,15^ This approach allowed us to examine the effects of complex and multidimensional cultural shifts through a quantitative lens, interpreting our data-driven results in the context of known medical movements.

The rise of evidence-based medicine may have been responsible for the greatest trends in our analysis, with large increases in the language of clinical trial designs. The language of causality has fallen out of favor, with cause-and-effect being replaced with risks and associations. This finding not only reflects changes in the discipline of research design but could suggest increased humility on the part of researchers in describing the significance of their work. With respect to patient centeredness, we observed a shift away from the individual patient to cohorts and populations of patients. As case reports have become less prevalent, articles have become more likely to describe patients in the plural form rather than focusing on an individual.

With respect to the role of patients in clinical trials, we observed a trend toward separating the entities of patient and disease. This trend was consistent across 4 of the 5 major medical journals that we assessed, in which we observed an overall increase in the proportion of titles that made use of a patient noun that is independent of a disease process. Whereas it was common in the 1970s and 1980s to describe *diabetic patients*^16^ or guidelines for *the diabetic*,^17^ we found no instances of the word *diabetic* being used as a noun or adjective describing a patient in *JAMA* from 2011 through 2015. Instead, the tendency has been to describe *patients with diabetes*^18^ or *adults with diabetes*.^19^ Representative examples of antimicrobial prophylaxis,^20,21^ hypertension,^22,23^ insulin therapy,^24,25^ radiotherapy,^26,27^ and corticosteroid therapy for asthma^28,29^ are shown in the Table. In this way, we argue that the trend has been to separate patients from disease and emphasize the patients rather than characterize them by their disease.

**Table.** Representative Journal Article Titles in *JAMA* in the Early vs Late Study Periods^a^

Topic	Representative Article	
	Early (1976-1980)	Late (2011-2015)
Antimicrobial prophylaxis	Erythromycin ointment for ocular prophylaxis of neonatal chlamydial infection (1980)^20^	Effect of fluconazole prophylaxis on candidiasis and mortality in premature infants: a randomized clinical trial (2014)^21^
Hypertension	Prazosin and clonidine for moderately severe hypertension (1978)^22^	Effect of aliskiren on progression of coronary disease in patients with prehypertension: the AQUARIUS randomized clinical trial (2013)^23^
Insulin therapy	Low- and high-dose intravenous insulin therapy for diabetic ketoacidosis (1979)^24^	Effect of sensor-augmented insulin pump therapy and automated insulin suspension vs standard insulin pump therapy on hypoglycemia in patients with type 1 diabetes: a randomized clinical trial (2013)^25^
Radiotherapy	Dianhydrogalactitol and radiation therapy: treatment of supratentorial glioma (1979)^26^	Association between radiotherapy vs no radiotherapy based on early response to VAMP chemotherapy and survival among children with favorable-risk Hodgkin lymphoma (2012)^27^
Corticosteroid therapy for asthma	Beclomethasone in steroid-dependent asthma: effective therapy and recovery of hypothalamo-pituitary-adrenal function (1977)^28^	Comparison of physician-, biomarker-, and symptom-based strategies for adjustment of inhaled corticosteroid therapy in adults with asthma: the BASALT randomized controlled trial (2012)^29^

^a^ Article titles in *JAMA* in the late period tend to explicitly state the experimental design and emphasize the patient as the subject of the title. In contrast, titles in the early period tend to use the therapeutic modality or disease entity as the subject, and typically do not use a noun that refers to the patient or patient group under study.

The trends that we observed in the role of patients in clinical research are best interpreted within the broader context of changes in the bioethics of research with human participants in the last 50 years. The 1960s were the start of serious academic discussion that began a transformation in bioethics and human subjects research, marked by Henry Beecher’s influential publication of “Ethics and Clinical Research” in 1966,^30^ which warned of the risks of unregulated human experimentation and encouraged researchers to reform.^31^ Beecher’s article directly resulted in the proliferation of federal and institutional regulations governing research with human participants,^32^ which began as early as February 1966 when the US Surgeon General requested the establishment of institutional review boards for human trials in hospitals and universities.^33^ However, it was not until after 1972, which marked the end of the widely condemned Tuskegee Syphilis Study, that major federal reform in the United States was enacted for research with human participants in the form of the National Research Act in 1974 and the National Commission for the Protection of Human Subjects. The Commission’s Belmont Report of 1979 continues to guide human subjects research today.^34,35^ Thus our 40-year study period beginning in the mid-1970s captures the period in which clinical investigators, beginning with efforts initiated in the 1960s, had begun to widely adopt the practice of research with human participants with explicit ethical guidelines.

Our decision to focus on the textual content of article titles allowed us to assess what authors and editors perceive to be the most important elements of the research being presented: the disease, the patient, the treatment, or some combination or subset thereof. The content of article titles consists of language and word choices that reflect underlying forces and processes currently operating in the culture of medicine, as well as the editorial guidelines and policies of the journal. In the findings of this study, several potential factors could account for changes in word choices and frequencies throughout a period, including conscious decisions by authors and editors, preferences driven by changing cultural norms in medicine, stylistic conventions driven by changing publication formats, increased academic interest in a research topic, and shifts in nomenclature. The extent of changes in editorial guidelines and policies as opposed to changes in the cultural norms within biomedical science is an important consideration in assessing the significance of our findings. Certainly, the content of article titles is determined in part by editorial policies that are owing to some combination of journal-specific values and wider journalistic conventions. With respect to *JAMA* and clinical research, an example is the journal’s adoption in the 1990s of a requirement to identify randomized trial designs in the title,^36^ in line with the Consolidated Standards of Reporting Trials (CONSORT) reporting guidelines.^37^

However, in terms our findings, particularly the shift to more patient-centric words in titles, we note several things. First, journal specific-values and wider journalistic conventions do not occur in a vacuum but are invariably influenced by the broader culture of medicine. Second, authors and journal editors typically come from the same academic or clinical disciples, and thus editors are likely to reflect the cultural norms of the larger group of authors. Third, the clinical reporting guidelines for randomized trials (CONSORT^37^), observational studies (Strengthening the Reporting of Observational Studies in Epidemiology [STROBE] reporting guidelines^38^), systematic reviews (Preferred Reporting Items for Systematic Reviews and Meta-analyses [PRISMA] reporting guidelines^39^), diagnostic/prognostic studies (Standards for Reporting Diagnostic Accuracy [STARD]^40^), and case reports (CARE^41^) do not explicitly recommend the use of patient-centric language in the title. In contrast, CONSORT recommends that randomized trials be identified in the title.^37^ Fourth, some changes in word frequency can certainly be attributed to specific changes in terminology, such as changes in diagnostic nomenclature. An example is illustrated in the eFigure in the Supplement, where the frequency of *renal failure* has decreased significantly and *kidney disease* has increased dramatically, a trend reflecting the more recent consensus-driven use of the phrase *chronic kidney disease*. However, none of the major trends that we discuss (eg, changing use of causal language and patient-centered nouns) can be merely attributed changes in diagnostic nomenclature. Finally, we observed similarly significant trends in 4 of 5 completely independent journals (Figure 3). These trends suggest that our findings of increased use of patient-centric words in titles are less likely to be due to isolated, independent, idiosyncratic editorial decisions, but instead reflect broader social and cultural processes.

### Limitations

This study has limitations. Our analysis only considered a 40-year period for 5 influential journals that speak to a general audience and thus may not be representative of less prestigious journals or journals with a narrower focus or a specialized audience. Future studies will therefore analyze a broader array of journals and involve earlier periods. In addition, our report focused only on an analysis of titles, which offers an important but limited view of the actual content and framing of an article. Interrogation of the titles of journal articles does not tell everything that might be learned about the thinking and motivations of authors and editors. Our study, however, provides a proof of principle that the approaches applied in this report hold promise for future analyses of longer sequences of words or larger portions of text (such as abstracts or discussion sections). These approaches can also be applied to the text of other article types (eg, review articles, consensus statements, editorials, and commentaries) and text sources (eg, conference proceedings, grand rounds, and continuing medical education) that would provide further insights into changing medical culture in different contexts. Because of our study design, the findings cannot be directly extrapolated to physician-patient interactions, although we might speculate that the language of biomedical literature mirrors how language is used in the clinical arena. The validity of the findings developed through our quantitative, data-driven analysis needs to be assessed in light of other social science studies that have documented the complex social history of biomedical research.^31,32,33,34,35^ We therefore believe that this quantitative text analysis complements other approaches, such as qualitative social science research and narrative medicine, that also enable our understanding of the relational aspects of medicine.

## Conclusions

The culture of medicine is undergoing continuous change, and the last 40 years have marked several significant paradigm shifts. We sought to uncover changing trends in medical language as a proxy for medical culture, using the wealth of embedded information contained within medical literature of the past 40 years. Using this approach, we identified trends in medical language that reflect the rise of evidence-based medicine, a shift in focus from individuals to populations, a separation of patient and disease, and a reemphasis on patients involved in clinical research. This data-driven analysis of medical language provides a unique window into the changing landscape of medical culture.

## Notes

Supplement. **eTable.** Inclusion and Exclusion Criteria for Defining the Presence of a Patient-Centric Noun **eFigure.** Frequency Trends of Selected Monogram and Bigrams
