# Supplementary material for: Trends in the Use of Common Words and Patient-Centric Language in the Titles of Medical Journals, 1976-2015
Source: JAMA Netw Open. 2019 Mar 22;2(3):e191083. doi: 10.1001/jamanetworkopen.2019.1083 (PMC6583309; doi:10.1001/jamanetworkopen.2019.1083)
Supplement: Supplement. — eTable. Inclusion and Exclusion Criteria for Defining the Presence of a Patient-Centric Noun eFigure. Frequency Trends of Selected Monogram and Bigrams [file jamanetwopen-2-e191083-s001.pdf]

Supplementary Online Content

Chen GM, Pather SR, DeLisser HM. Trends in the use of common words and patient-centric language in the titles of medical journals, 1976-2015. *JAMA Netw Open*. 2019;2(3):e191083. doi:10.1001/jamanetworkopen.2019.1083

eTable. Inclusion and Exclusion Criteria for Defining the Presence of a Patient-Centric Noun

eFigure. Frequency Trends of Selected Monogram and Bigrams

This supplementary material has been provided by the authors to give readers additional information about their work.

© 2019 Chen GM et al. *JAMA Network Open.*

eTable. Inclusion and Exclusion Criteria for Defining the Presence of a Patient-Centric Noun

Inclusion criteria	Exclusion criteria
• Use of a singular or plural noun referring to a patient or patients anywhere in the title<br>• Presence of a nouns that describe a human being based on their personhood or gender (e.g. man, woman, patient, individual)<br>• Presence of a noun that describes an individual and their role in the healthcare system<br>• Example words: patients, adults, man/men, woman/women, persons, adolescents, infants, neonate, newborn, people, resident, a person with disease X, veterans, donor, recipient, individual, participant, family member, survivor, neonate, newborn.	• Nouns describing healthcare providers in that role: e.g. nurse aide, nursing assistant, doctor, provider, physician, etc.<br>• Nouns referring to a patient that characterizes them by their disease (addict, diabetic) or disease risk factor (the elderly), race or ethnicity (African American, but do not exclude African American man)<br>• Description of a patient as a research entity, e.g. “subject”<br>• Population (e.g. elderly population, adult population)<br>• Use of a patient ‘noun’ in adjective form (e.g. patient outcomes, infant outcomes, infant populations

Raters classified each title as patient-centric if it satisfied one or more inclusion criteria and none of the exclusion criteria. Discrepancies were resolved by consensus with the senior author.

© 2019 Chen GM et al. *JAMA Network Open.*

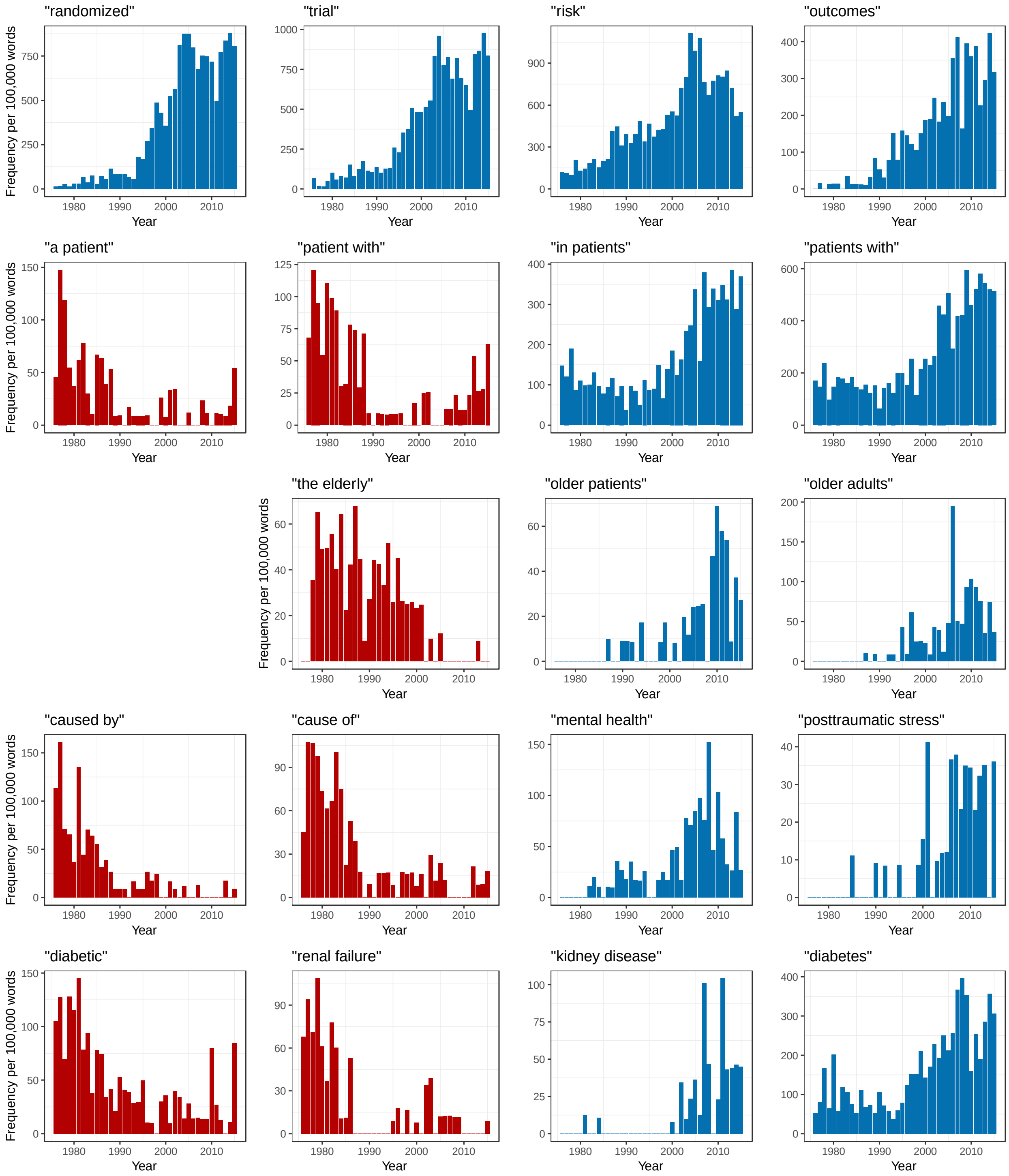

eFigure. Frequency Trends of Selected Monogram and Bigrams

Each bar represents the frequency per 100,000 monograms or bigrams in each one-year period.

© 2019 Chen GM et al. *JAMA Network Open.*
